# Investigating contact toxicity of *Geranium* and *Artemisia* essential oils on *Bemisia tabaci* Gen.

**Published:** 2013

**Authors:** Fatemeh Yarahmadi, Ali Rajabpour, Nooshin Zandi Sohani, Leila Ramezani

**Affiliations:** 1 *Department of Plant Protection, Ramin Agricultural and Natural Resources University, I.R. Iran*

**Keywords:** Greenhouse Cucumber, Essential Oils, *Pelargonium roseum*, *Artemisia sieberi*, Organic Product, Potato Whitefly, Sweet

## Abstract

**Objective:** Sweet potato whitefly, *Bemisia tabaci* Gen. (*B. tabaci*), is one of the most important pests of various greenhouse crops in Iran. Nowadays, chemical insecticides are broadly used for control of the pests that causes risk to consumer's health. For the first time, contact toxicity of *Pelargonium roseum* Andrews and *Artemisia sieberi* Besser essential oils on *B. tabaci* and its possible application against the whitefly was evaluated in 2012.

**Materials and Methods: **Essential oil with concentrations of 2500, 1250, 125, and 12 ppm were used. Infested leaves of greenhouse cucumber were treated by mentioned concentrations. After 24 hours, mortality of *B. tabaci* was recorded and compared after correcting by Abbot's formula.

**Results: **Results showed that all concentrations of the essential oil could significantly reduce population of *B. tabaci* compared with the control treatment. Phytotoxicity of the treated leaves were recorded after 24, 48, and 72 hours and compared with the control. Concentrations of 2500, 1250, and 125 ppm caused severe phytotoxicity on greenhouse cucumber leaves and therefore are not suitable for greenhouse application. Phytotoxicity of 12 ppm was relatively low.

**Conclusions: **This data implicated suitable protective effects of the essential oils to the pest infestation. Therefore, essential oils distillated from *Geranium* and *Artemisia* could be applied to control *B. tabaci* in greenhouse cucumber at V/V 12 ppm.

## Introduction

*Bemisia tabaci *(Gennadius), sweet potato whitefly, is a polyphagous and multivoltine insect pest responsible for high economic losses in many crops (Valle Piherio et al., 2009 ▶; Khanjani, 2007 ▶). It is also a major pest of greenhouse crops, particularly ornamentals, although major crops under greenhouse production such as tomato, pepper, beans, eggplant, and cucumber are also attacked (Mound, 1978 ▶; Cock, 1986 ▶). *B. tabaci* was first reported for causing damage to greenhouse ornamentals in the United States (Price et al., 1986 ▶). Since invasion of sweet potato whitefly into Iran in 1934, it has caused serious losses to cotton, soybean, watermelon, muskmelon, various vegetables, and ornamentals (Omid Bakhsh et al., 2010 ▶). The impact of direct feeding and honeydew excreta that favors sooty mold production are factors that affect crop yield in both quantitative and qualitative terms (Oliviera et al., 2001 ▶). *B. tabaci* transmits plant viruses in seven distinct virus groups including: geminiviruses, closteroviruses, carlaviruses, potyviruses, nepoviruses, luteoviruses and a DNA-containing rod-shaped virus (Thompson, 2011 ▶). Control of *B.tabaci *populations worldwide is primarily dependent on repeated applications of conventional insecticide.

Although effective, their repeated use for decades has disrupted natural biological control systems and led to resurgence of this insect (Dittrich et al., 1990 ▶), sometimes resulted in the development of resistance (Roditakis et al., 2005 ▶), had undesirable effects on non-target organisms, and fostered environmental and human health concerns (Hayes and Laws, 1991 ▶). Because of the problems associated with the use of synthetic pesticides researchers look for natural plant protection compounds such as botanicals, insecticides, and antifeedants (Isman, 2006 ▶). Natural products are an excellent alternative to synthetic pesticides as a means to reduce negative impacts to human health and the environment (Koul et al., 2008 ▶). Plant essential oils may be an alternative source for *B. tabaci control* because these are secondary metabolites and source of bioactive chemicals that plants produce for defense against herbivory and disease source of bioactive chemicals (Suthisut et al., 2011 ▶). Iran has rich medical flora and essential oils from Iranian plants and their major constituents show potential for utilization in insect pest management programs due to their availability, efficiency, and safety to environment and non-target organisms (Ebdollahi, 2011 ▶). This research describes a study aimed at assessing the potential of essential oils of *Artemisia siebberi* Besser (Asteraceae) and *Geranium roseum* Andrews (Geraniaceae) for use as commercial insecticides. 

## Materials and Methods

**Host plants**

Greenhouse cucumber *Cucumis sativus* L. (Var. Negeen) used as the host plant. Greenhouse cucumber comprises 90% of greenhouse vegetable crops of Iran (Baniameri and Nasrollahi, 2003). The plants were planted in plastic pots (15 cm diameter and 25 cm height). Field soil (Sand: soil and humus with same ratio) used for plant cultures.

**Insect rearing**

Rearing of *B. tabaci* was carried out in insectarium at temperature 26±3 ^°^C and photoperiod 14:10 (light: dark). The plant pots were carried to cages (60×60×120 cm). Lateral sides of the cages were covered by thin gauze (10×10) mesh led to suitable ventilation. The whiteflies were collected from Khuzestan's cucumber greenhouses. Whitefly cultures were established by transferring 50-60 adults to each cage. The insects were screened on the upper surface of the plants using circular clips cages (2 cm in diameter, 3.14 surface area) for 72 hours.

**Essential oil bioassay**

*Artemisia sieberi *and *Geranium roseum *essential oils were purchased from Barij Esans Company, Kashan, Iran. **Different concentrations of the essential oils, 2500, 1250, 125, and 12 ppm, were prepared. **The developmental stages that were treated in toxicity experiments consisted of 5 d-old eggs, 2 d-old first-instar nymphs, and 2 d-old pupae. To achieve the developmental stages, 10 *B. tabaci *females were released into each clip cage placed under the detached cucumber leaf for 24 hours. After this period, all whiteflies were removed. The developmental stages of 5 d-old eggs, 2 d-old first-instar nymphs, and 2 d-old pupae were obtained 5, 9, and 16 days after removal of the adults, respectively. The number of B. tabaci individuals was counted on each test leaf before the treatment. Each experiment had 4 replications. Leaves with mentioned developmental stage were dipped in the essential oil concentration for 5 seconds (Yang et al., 2010 ▶). Mortalities were recorded after 12, 24, and 48 hours. The phytotoxicity on cucumber leaves (due to the essential oil) was recorded at 24, 48, and 72 hours after the treatment. All trials were carried out in an insectarium at temperature of 26±3 ^°^C and a photoperiod of 14:10 (Light: dark).

**Statistical analyses**

Mortality percentage was determined. Analysis of Variance (ANOVA) was performed using SPSS software Version 16 (SPSS Inc., Chicago, USA) to compare mortality in different treatments. ANOVA was followed by a post-hoc Duncan’s test (p<0.05 as significant).

## Results

Mortality percentages of the whitefly life stages in *Geranium* and *Artemisia* treatments were showed in Tables 1 and 2, respectively. ANOVA indicated that all concentrations of both essential oils were significantly suppressed all developmental stages of B. tabaci 12 hours after the treatment (df=89, 351; F=4.23; p=0.00). 

**Table 1:** Mean of observed B. tabaci mortality and cucumber leaves phytotoxicity in different treatments at various times.

**Life stage**	**Duration after the treatment**	**The essential oil concentrations (PPM)**				
		**2500**	**1250**	**125**	**12**	**Control**
**Egg**	12 h	93 ab	95 ab	82 b	63 bc	0 d
	24 h	100 a	100 a	98 ab	96 ab	5 c
	48 h	100 a	100 a	100 a	98 ab	10 c
**Nymph**	12 h	96 ab	92 a	90 ab	74 ab	0 d
	24 h	100 a	100 a	100 a	100 a	5 c
	48 h	100 a	100 a	100 a	100 a	5 c
**Pupae**	12 h	91 ab	92 ab	85 ab	66 bc	0 d
	24 h	98 ab	100 a	100 a	92 ab	0 d
	48 h	100 a	100 a	100 a	96 ab	0 d
**Phytotoxicity**	24 h	72 b	31 bc	27 bc	0 d	0 d
	48 h	89 b	43 bc	36 bc	5 c	0 d
	72 h	100 a	75 b	50 bc	5 c	0 d

Means followed by the same letter are not significantly different (p<0.05,Duncan’s test).

**Table 2:** Mean of observed B. tabaci mortality and cucumber leaves phytotoxicity in different treatments at various times in *Artemisia* treatment

**Life stage**	**Duration after the treatment**	**The essential oil concentrations (PPM)**				
		**2500**	**1250**	**125**	**12**	**Control**
**Egg**	12 h	95 ab	96 ab	73 b	69 bc	0 d
	24 h	100 a	100 a	100 ab	98 ab	0 d
	48 h	100 a	100 a	100 a	100 ab	3 c
**Nymph**	12 h	91 ab	95 ab	83 ab	70 b	0 d
	24 h	100 a	100 a	100 a	98 a	0 d
	48 h	100 a	100 a	100 a	100 a	5 c
**Pupae**	12 h	93 ab	90 ab	81 ab	65 bc	0 d
	24 h	98 ab	100 a	100 a	90 ab	0 d
	48 h	100 a	100 a	100 a	95 ab	0 d
**Phytotoxicity**	24 h	61 b	39 bc	18 bc	0 d	0 d
	48 h	89 b	53 bc	29 bc	3 c	0 d
	72 h	97 a	61 b	46 bc	7 c	0 d

Means followed by the same letter are not significantly different (p<0.05, Duncan’s test)

No significant differences were observed between different concentrations of the essential oils. However, percentage of phytotoxicity was significantly different among them (df=29, 111; F=3.35; p=0.00). Results showed that mortality percentage of B. tabaci* and* phytotoxicity on cucumber leaves increased during the time of the experiment. The least *phytotoxic effects *of two essential oils* were *o*bserved* at 12 ppm solution. 

Phytotoxicity in cucumber were too high at 2500, 1250, and 25 ppm. Therefore, the concentrations could not be applied to control *B. tabaci* on greenhouse cucumber. Concentration of 12 ppm was suitable for controlling B. tabaci on greenhouse cucumber because of its appropriate effect on all developmental stages of sweet potato whitefly and its relatively low phytotoxicity. 

## Discussions

Essential oils or constituents from certain plants have a broad spectrum of activities against pests, such as *B. tabaci* because many of them are selective to pests, and have little or no harmful effects on non-target organisms and the environment (Isman, 2000 ▶). Suitable contact and fumigant toxicities of some plant essential oils were reported by researchers. 

High suppression of *B. tabaci* population was reported by some essential oils such as thyme and garlic (Kim et al., 2011 ▶). Essential oil vapors from *Satureja hortensis* L., *Ocimum basilicum* L., and *Thymus vulgaris* L. (Lamiacae) had high toxicities against the nymphs and adults of *Tetranychus urticae* Koch (Acari: Tetranychidae) and adults of *B. tabaci* (Aslan et al., 2004 ▶). 

Effectiveness of *Petiveria alliacea* L. (Phytolaccaceae) essential oil against *Trialeurods vaporariorum* L. was proven on tomato in laboratory and greenhouse conditions (Garcio et al., 2007 ▶). Present results about high contact toxicity of essential oil are in accordance with those presented by Yang et al. (2010) ▶. They found high contact toxicities of *Thymus vulgaris* L., *Pogostemon cablin* Blanco, and *Corymbia citriodora* Hook essential oils against *B. tabaci* biotype B. on greenhouse tomato. However, our result about effect of the essential oil on greenhouse cucumber conflicts with those observed by Yang et al. (2010) ▶. The conflicting results may be due to the different essential oil and host plants. 

Considering other control strategies of pests, both efficiency and being environmental friendly reasons makes essential oils much preferable insecticides against different pest groups, particularly against greenhouse pests such as whiteflies. Recent studies showed that compared with other control strategies, essential oil applications have several advantages. Their applications affect whiteflies and some other pest in a short time by killing all developmental stages. Using the essential oils as insecticides is also safer for the environment and human health because of their low toxicity and shorter degradation time.
